# Nicorandil Attenuates Monocrotaline-Induced Vascular Endothelial Damage and Pulmonary Arterial Hypertension

**DOI:** 10.1371/journal.pone.0033367

**Published:** 2012-03-30

**Authors:** Makoto Sahara, Masataka Sata, Toshihiro Morita, Yasunobu Hirata, Ryozo Nagai

**Affiliations:** 1 Department of Cardiovascular Medicine, University of Tokyo Graduate School of Medicine, Tokyo, Japan; 2 Department of Cardiovascular Medicine, Institute of Health Biosciences, The University of Tokushima Graduate School, Tokushima, Japan; 3 Department of Cardiology/Cardiovascular Research Center, Massachusetts General Hospital, Harvard Medical School, Boston, Massachusetts, United States of America; Istituto Dermopatico dell'Immacolata, Italy

## Abstract

**Background:**

An antianginal K_ATP_ channel opener nicorandil has various beneficial effects on cardiovascular systems; however, its effects on pulmonary vasculature under pulmonary arterial hypertension (PAH) have not yet been elucidated. Therefore, we attempted to determine whether nicorandil can attenuate monocrotaline (MCT)-induced PAH in rats.

**Materials and Methods:**

Sprague-Dawley rats injected intraperitoneally with 60 mg/kg MCT were randomized to receive either vehicle; nicorandil (5.0 mg·kg^−1^·day^−1^) alone; or nicorandil as well as either a K_ATP_ channel blocker glibenclamide or a nitric oxide synthase (NOS) inhibitor *N* ^ω^-nitro-l-arginine methyl ester (l-NAME), from immediately or 21 days after MCT injection. Four or five weeks later, right ventricular systolic pressure (RVSP) was measured, and lung tissue was harvested. Also, we evaluated the nicorandil-induced anti-apoptotic effects and activation status of several molecules in cell survival signaling pathway in vitro using human umbilical vein endothelial cells (HUVECs).

**Results:**

Four weeks after MCT injection, RVSP was significantly increased in the vehicle-treated group (51.0±4.7 mm Hg), whereas it was attenuated by nicorandil treatment (33.2±3.9 mm Hg; *P*<0.01). Nicorandil protected pulmonary endothelium from the MCT-induced thromboemboli formation and induction of apoptosis, accompanied with both upregulation of endothelial NOS (eNOS) expression and downregulation of cleaved caspase-3 expression. Late treatment with nicorandil for the established PAH was also effective in suppressing the additional progression of PAH. These beneficial effects of nicorandil were blocked similarly by glibenclamide and l-NAME. Next, HUVECs were incubated in serum-free medium and then exhibited apoptotic morphology, while these changes were significantly attenuated by nicorandil administration. Nicorandil activated the phosphatidylinositol 3-kinase (PI3K)/Akt and extracellular signal-regulated kinase (ERK) pathways in HUVECs, accompanied with the upregulation of both eNOS and Bcl-2 expression.

**Conclusions:**

Nicorandil attenuated MCT-induced vascular endothelial damage and PAH through production of eNOS and anti-apoptotic factors, suggesting that nicorandil might have a promising therapeutic potential for PAH.

## Introduction

Pulmonary arterial hypertension (PAH) is a progressive fatal disorder with a poor prognosis [1], [2]. The changes in the pulmonary vasculature in PAH involve persistent vasoconstriction, vascular smooth muscle cell proliferation, and thrombosis [1]–[3]. Although the exact pathogenesis of PAH is still uncertain, it is thought that vascular endothelial damage and dysfunction play a crucial role in triggering pathological vascular remodeling [4]. In addition, experimental studies suggest that endothelial cell apoptosis in pulmonary microvasculature causes arteriolar occlusion and increases pulmonary vascular resistance [4]–[7], suggesting that vascular endothelial cell apoptosis is closely associated with the pathogenesis of PAH.

Nicorandil is a unique hybrid vasodilator and exerts 2 vasodilator actions; adenosine triphosphate (ATP)-sensitive potassium (K_ATP_) channel opening and nitric oxide (NO) release [8]. Nicorandil is not only an antianginal drug but also exerts cardioprotective effects on the ischemic myocardium due to its K_ATP_ channel-opening action, thereby mimicking the phenomenon of ischemic preconditioning [9]. Cumulative evidence suggests that nicorandil has several beneficial effects on the cardiovascular system; however, its effects on the pulmonary vasculature under PAH remain undetermined.

On this basis, we evaluated the efficacy of nicorandil in an experimental rat PAH model induced by monocrotaline (MCT). Also, the mechanisms of action of nicorandil were investigated in vivo and in vitro. Here, we show that nicorandil attenuates MCT-induced endothelial damage and apoptosis in pulmonary vasculature under PAH through production of endothelial NO synthase (eNOS) and anti-apoptotic factors; the production is mediated by the cell survival signaling cascades, phosphatidylinositol 3-kinase (PI3K)/Akt and extracellular signal-regulated kinase (ERK) pathways, which are mainly activated via the opening of K_ATP_ channels. The results suggest that nicorandil may have a therapeutic potential for PAH.

## Materials and Methods

### Animals

Wild-type Sprague-Dawley rats were purchased from Japan SLC. All experimental procedures and protocols were approved by the Institutional Committee for Animal Research at the University of Tokyo (#1621T 132) and complied with the Guide for the Care and Use of Laboratory Animals (National Institutes of Health (NIH) publication no. 86-23; revised 1985).

### The animal model of PAH and experimental protocols

Eight-week-old male Sprague-Dawley rats were injected intraperitoneally with saline (control) or 60 mg/kg MCT (Wako). In the prevention protocol, the rats injected with MCT were randomized to receive either a vehicle, nicorandil (2.5–7.5 mg·kg^−1^·day^−1^) alone, or nicorandil (5.0 mg·kg^−1^·day^−1^) with either 5.0 mg·kg^−1^·day^−1^ of the K_ATP_ channel blocker glibenclamide or the NO synthase (NOS) inhibitor *N* ^ω^-nitro-l-arginine methyl ester (l-NAME; 1 mg/mL in drinking water) from immediately after the MCT injection. The vehicle, nicorandil, and glibenclamide were administered continuously by an implanted subcutaneous osmotic pump (Alzet; Durect Corporation). Separately, one group of the MCT-injected rats was administered a pan-caspase inhibitor, Z-Val-Ala-Asp(OMe)-CH_2_F (ZVAD-fmk; R&D Systems), as a bolus into the tail vein 4 times (first immediately after the MCT injection and 1, 3, and 7 days later; total dose, 3.3 mg/kg). In addition, the other group of the MCT-injected rats was treated with a NO donor, sustained release isosorbide dinitrate (sr-ISDN), by oral administration at a dose rate of either 10, 50, or 100 mg·kg^−1^ once a day. Each group comprised 8–10 rats. The right ventricular systolic pressure (RVSP) of the rats was measured by inserting polyethylene catheters into the right ventricle 28 days after the MCT injection. The rats were then euthanized, and the hearts and lungs were harvested. The weight ratio of the right ventricle (RV) to left ventricle (LV) including the septum (RV/LV ratio) was determined. The right lungs were fixed in methanol or 4% paraformaldehyde and embedded in paraffin for histological analysis. The left lung segments were snap frozen in liquid nitrogen for western blotting.

In the reversal protocol, the rats were injected with saline (control) or 60 mg/kg MCT, and 21 days later, the rats were randomized to receive either the vehicle, nicorandil (5.0 mg·kg^−1^·day^−1^) alone, or nicorandil with glibenclamide or l-NAME. Two weeks after the initiation of treatment, the RVSP was measured, and the hearts and lungs were harvested for analyses.

### Survival Analysis

In a separate experiment, we examined the effects of nicorandil on the survival of the MCT-injected rats in both the prevention and reversal protocols. The day of the MCT injection was defined as day 0, and the survival analysis observation continued up to day 42. The groups in both protocols comprised 12–15 rats.

### Histological analysis

The paraffin-embedded sections were processed for hematoxylin and eosin, elastic Van Gieson, and immunohistochemical staining for examination under light microscopy. The medial wall thickness of the pulmonary arterioles (PAs) was calculated and expressed as follows: %medial wall thickness = ([medial thickness×2]/external diameter)×100. Immunohistochemical analyses involved the incubation of the sections with the primary antibodies (anti-α-smooth muscle actin [αSMA], Sigma; anti-CD68, Serotec; and anti-eNOS, BD Biosciences), followed by incubation with a biotinylated secondary antibody (Dako) using the avidin-biotin complex technique with Vector Red substrate (Vector Laboratories). The nuclei were counterstained with hematoxylin.

### Terminal deoxynucleotidyl transferase-mediated 2′-deoxyuridine 5′-triphosphate nick-end labeling staining

To detect apoptotic cell death, terminal deoxynucleotidyl transferase-mediated 2′-deoxyuridine 5′-triphosphate (dUTP) nick-end labeling (TUNEL) staining with immunofluorescence staining for αSMA was performed using an in situ death detection kit (Roche Molecular Biochemicals), as previously described [10]. Using a confocal microscope (FLUOVIEW FV300; Olympus), the number of PAs (external diameter, 20–100 µm) having TUNEL-positive endothelial cell(s) was counted in each lung cross section, and the percentage was calculated as follows: ([the number of PAs with TUNEL-positive endothelial cell(s)]/[a total number of equally sized PAs (external diameter, 20–100 µm)]×100.

### Immunohistochemical staining for Ki67 expression

To assess proliferation of smooth muscle cells (SMCs) in the media of PAs, immunofluorescent double staining of lung frozen sections for Ki67 and αSMA was performed. After blocking with 1% bovine serum albumin and 5% goat serum in PBS, the sections were incubated overnight with anti-Ki67 rabbit antibody (Abcam) and anti-αSMA mouse antibody (Sigma), followed by incubation with Alexa Fluor 488-conjugated anti-rabbit IgG and Alexa Fluor 594-conjugated anti-mouse secondary antibodies (Molecular Probes) for 1 h. After washing, the nuclei were counterstained with DAPI (Sigma) before mounting and imaging by a confocal microscope. The number of proliferating PA-SMCs with Ki67 positive nuclei was expressed as the percentage of Ki67-positive cells over the total number of αSMA-positive SMCs in the media of 30–40 PAs (external diameter, 20–100 µm) per rat.

### Cell culture

Human umbilical vein endothelial cells (HUVECs) were purchased from Cambrex BioScience and were cultured in endothelial growth medium-2 (EGM-2) (Clonetics) containing 2% fetal bovine serum (FBS) and growth factors. HUVECs were grown to 80–90% confluence and were then starved in serum-free medium. After 4 h, the serum-starved HUVECs were stimulated with several agents; including nicorandil (10–1000 µmol/L); a K_ATP_ channel opener, diazoxide (100 µmol/L; Chugai); or nicorandil (100 µmol/L) with either glibenclamide (10 µmol/L), l-NAME (2 mmol/L), a PI3K inhibitor, LY294002 (10 µmol/L; Calbiochem), or an ERK-specific mitogen-activated protein kinase kinase (MEK) inhibitor, PD98059 (10 µmol/L; Calbiochem); for the indicated time intervals.

### Analyses of cell morphology, viability, and apoptosis

HUVECs were incubated in a serum-free medium with or without nicorandil for 48 h. The cells were then observed under a phase contrast microscope (Olympus). Cell viability was determined using 3-(4,5-dimethylthiazol-2-yl)-5-(3-carboxymethoxyphenyl)-2H-tetrazolium inner salt assay (MTS; Promega Corporation), and the percent cell death was calculated as follows: 100×{1−[viability of treated (serum-starved) endothelial cells/viability of untreated endothelial cells]}. In a separate series, HUVECs that were incubated in serum-free medium for 12 h were subjected to TUNEL staining to detect apoptotic cell death, according to the manufacturer's instructions. The nuclei were counterstained with Hoechst 33258 (Sigma). TUNEL-positive nuclei were counted in 10 randomly selected fields using a confocal microscope (Olympus) and were expressed as a percentage of the total number of nuclei.

### Western blotting

Proteins were extracted from the lung tissues or HUVECs after homogenization in a lysis buffer containing a protease inhibitor cocktail (Sigma). The protein samples (5–10 µg) were separated by sodium dodecyl sulfate-polyacrylamide gel electrophoresis (SDS-PAGE) and transferred onto a polyvinylidene fluoride membrane (Hybond-P; GE Healthcare). The membranes were incubated with primary antibodies to eNOS, cleaved caspase-3, Akt, Ser473-phospho-Akt, ERK, Thr202/Thr204-phospho-ERK1/2, Bad, Ser112-phospho-Bad (Cell Signaling Technology), and Bcl-2 (BD Biosciences), followed by incubation with a horseradish peroxidase-conjugated secondary antibody. Next, an enhanced chemiluminescence system (ECL Plus; GE Healthcare) was used to detect immunoblotting, and bands were visualized and quantified with a lumino-analyzer (LAS-1000; Fujifilm). The signal intensity was normalized to β-actin expression.

### Akt kinase assay

Akt kinase activity was determined in the protein (20 µg) extracted from the HUVEC lysates by detecting the phosphorylated glycogen synthase kinase (GSK)-3 fusion protein with the Akt kinase assay kit (Cell Signaling Technology), according to the manufacturer's instructions.

### Statistical analysis

Data are presented as the mean ± standard deviation (SD). The comparison of the means was performed by a one-way analysis of variance (ANOVA) followed by Scheffé's post hoc test. The survival curves were analyzed by the Kaplan-Meier method and compared using the Wilcoxon rank sum test. Statistical significance was defined as *P*<0.05.

## Results

### Nicorandil prevents the progression of MCT-induced PAH

**Figure 1:**
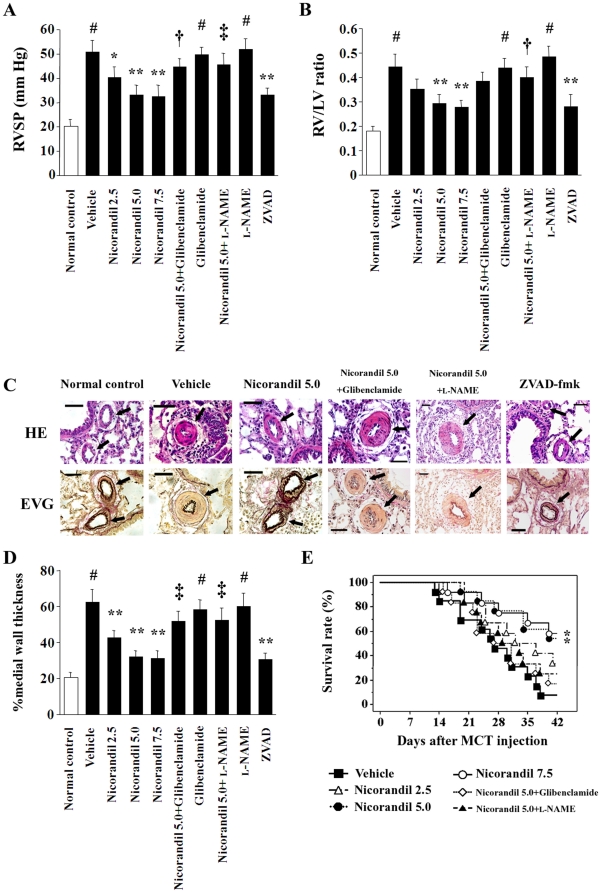
The effects of nicorandil in the prevention protocol. The RVSP (**A**) and the RV/LV ratio (**B**) 28 days after MCT injection are shown. Treatment with nicorandil and ZVAD-fmk attenuated the MCT-induced increase in both these parameters, while these effects of nicorandil were blocked by glibenclamide and l-NAME. Each group comprised 8–10 rats. ^#^ *P*<0.01 vs. normal control; ^*^ *P*<0.05 and ^**^ *P*<0.01 vs. vehicle; **^†^** *P*<0.05 and **^‡^** *P*<0.01 vs. nicorandil (5.0 mg·kg^−1^·day^−1^). (**C**) Histological findings of the PAs (arrows). Top, hematoxylin and eosin (HE) staining; bottom, elastic Van Gieson (EVG) staining. Scale bar, 50 µm. (**D**) MCT markedly increased the percent medial wall thickness of the PAs (#), and nicorandil and ZVAD-fmk attenuated MCT-induced medial wall thickening (**). In contrast, the effects of nicorandil were inhibited by glibenclamide and l-NAME (**‡**). (**E**) Survival analysis in the prevention protocol. Each group comprised 12–13 rats. ^*^ *P*<0.05 vs. vehicle.

The RVSP in the vehicle-treated group in the prevention protocol was significantly higher than that of the normal controls at 28 days after the MCT injection (51.0±4.7 mm Hg vs. 20.2±2.8 mm Hg; *P*<0.01) (Figure 1A). Nicorandil attenuated the MCT-induced increase in RVSP in a dose-dependent manner, and the RVSP was 33.2±3.9 mm Hg in rats treated with 5.0 mg·kg^−1^·day^−1^ nicorandil (*P*<0.01 vs. the vehicle). The effect of nicorandil was markedly inhibited by glibenclamide (44.8±3.4 mm Hg) and l-NAME (45.6±4.8 mm Hg; *P*<0.05 vs. nicorandil alone). The pan-caspase inhibitor ZVAD-fmk also attenuated the MCT-induced increase in RVSP (33.0±3.1 mm Hg; *P*<0.01 vs. the vehicle). Further, the RV/LV ratio, which was increased in the vehicle group, was also significantly attenuated in the nicorandil- and ZVAD-fmk-treated groups (Figure 1B). The systemic blood pressure and heart rate of the rats did not vary among the groups (data not shown).

Histological analysis revealed that the percent medial wall thickness of the PAs in the vehicle-treated group was significantly greater than that of the normal controls (62.7±7.0% vs. 20.5±2.9%; *P*<0.01) (Figure 1C and 1D). Treatment with 5.0 mg·kg^−1^·day^−1^ nicorandil (32.0±3.4%) and ZVAD-fmk (30.6±3.6%) attenuated the MCT-induced medial wall thickening (*P*<0.01 vs. the vehicle, respectively); however, these effects of nicorandil were inhibited by glibenclamide and l-NAME.

The survival analysis revealed that nicorandil significantly improved the survival rate in the MCT-injected rats in the prevention protocol (Figure 1E). The survival rates at 28 and 42 days after the MCT injection were 46% and 8% in the vehicle group, and those were increased to 77% and 54% in the nicorandil-treated group (5.0 mg·kg^−1^·day^−1^; *P*<0.05), respectively, whereas the coadministration of glibenclamide or l-NAME diminished the effect of nicorandil (Figure 1E).

In a separate series, the rats with MCT-induced PAH were treated with a slow NO-releasing drug, sr-ISDN. Low and middle doses of sr-ISDN (10 or 50 mg·kg^−1^·day^−1^) attenuated the MCT-induced increase in RVSP (41.8±4.3 or 38.6±3.4 mm Hg; *P*<0.05 vs. the vehicle) without changing systemic blood pressure and heart rate, although the decreasing degree in RVSP was modest compared with 5.0–7.5 mg·kg^−1^·day^−1^ nicorandil. High dose of sr-ISDN (100 mg·kg^−1^·day^−1^) attenuated the MCT-induced increase in RVSP to a greater degree (35.8±4.0 mm Hg; *P*<0.01 vs. the vehicle); however, high dose of sr-ISDN also significantly decreased systemic blood pressure in the rats with MCT-induced PAH (74.4±8.8 mm Hg vs. 91.8±8.6 mm Hg in the vehicle; *P*<0.01) and, probably due to the induction of severe hypotension, did not improve the survival rate in the MCT-injected rats (data not shown).

### Nicorandil improves the histopathological findings in MCT-injured lungs

**Figure 2:**
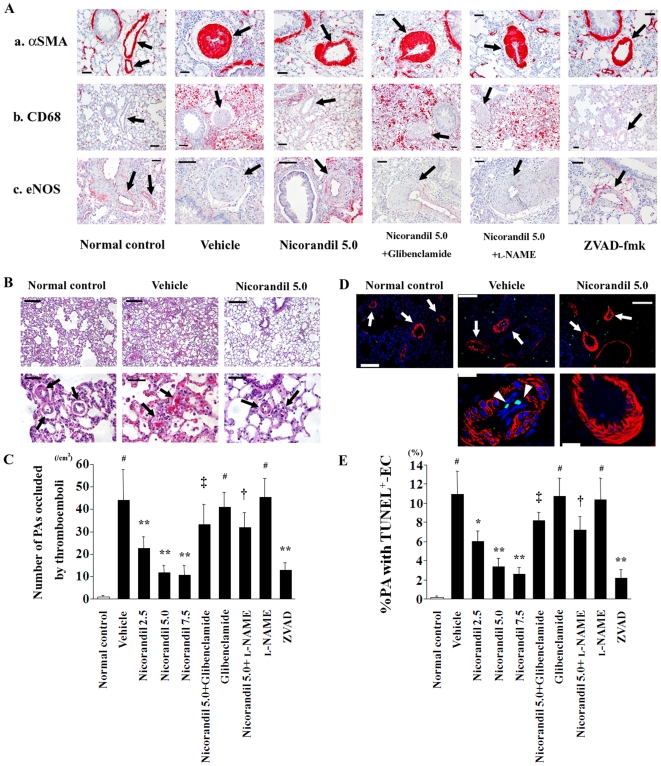
Nicorandil improves the histopathological findings of MCT-injured lungs. (**A**) Immunohistochemical staining for αSMA (**a**), CD68 (**b**), and eNOS (**c**) in lung sections are shown. Histopathologically, MCT injury induced the thickened medial wall of PAs (arrows) that was composed of αSMA-positive cells, the recruitment of CD68-positive macrophages into the perivascular areas, and the deficiency of eNOS expression in the endothelium of the pulmonary vasculature (*see* vehicle). Nicorandil and ZVAD-fmk improved these deleterious histopathological changes in the MCT-injured lungs, whereas glibenclamide and l-NAME blocked the effects of nicorandil. Scale bar, 50 µm. (**B**) MCT readily induced thromboemboli formation that occluded the small PAs (arrows; *see* vehicle), which was attenuated by nicorandil. HE staining. Scale bars, 100 µm (top) and 50 µm (bottom). (**C**) The number of small PAs that were occluded by thromboemboli was counted in the lung sections. (**D**) TUNEL staining with immunofluorescence staining for αSMA in lung sections. MCT readily induced endothelial cell apoptosis (arrowheads) in a number of PAs (arrows; *see* vehicle), which was attenuated by nicorandil. TUNEL (green); αSMA (red); and nuclei (blue). Scale bars, 100 µm (top) and 20 µm (bottom). (**E**) The number of small PAs having TUNEL-positive endothelial cell(s) [EC(s)] was counted in lung sections, and the proportion of PAs with TUNEL-positive EC(s) in a total of equally sized PAs was calculated. ^#^ *P*<0.01 vs. normal control; ^*^ *P*<0.05 and ^**^ *P*<0.01 vs. vehicle; **^†^** *P*<0.05 and **^‡^** *P*<0.01 vs. nicorandil (5.0 mg·kg^−1^·day^−1^).

**Figure 3:**
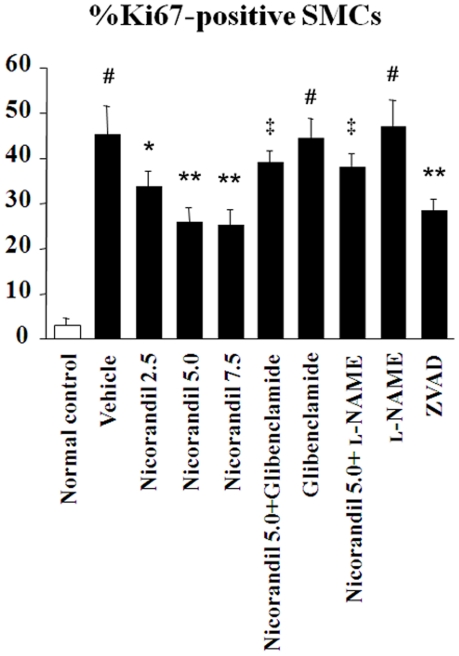
Nidorandil attenuates proliferation of PA-SMCs in MCT-injured lungs. Immunofluorescent double staining of lung frozen sections for Ki67 and αSMA was performed. The number of proliferating PA-SMCs with Ki67 positive nuclei expressed as the percentage of Ki67-positive cells over the total number of αSMA-positive SMCs in the media of 30–40 PAs (external diameter, 20–100 µm) per rat was significantly reduced by treatment with nicorandil and ZVAD-fmk. In contrast, glibenclamide and l-NAME diminished the effect of nicorandil, respectively. ^#^ *P*<0.01 vs. normal control; ^*^ *P*<0.05 and ^**^ *P*<0.01 vs. vehicle; **^‡^** *P*<0.01 vs. nicorandil (5.0 mg·kg^−1^·day^−1^).

Immunohistochemical findings revealed that nicorandil and ZVAD-fmk attenuated both the thickening of the PA's media that was composed of αSMA-positive cells and the recruitment of macrophages into the perivascular areas in MCT-injured lungs (Figure 2A). MCT markedly impaired eNOS expression in the endothelium of the pulmonary vasculature, and the expression was restored by treatment with nicorandil and ZVAD-fmk (Figure 2A). As shown in Figure 2B and 2D, MCT readily induced thromboemboli formation and endothelial cell apoptosis in small PAs (external diameter, 20–100 µm), and nicorandil and ZVAD-fmk attenuated these deteriorated changes (Figure 2B–2E). Treatment with nicorandil also significantly reduced the number of proliferating SMCs with Ki67 positive nuclei in the media of remodeled PAs (Figure 3). In contrast, glibenclamide and l-NAME blocked these effects of nicorandil, respectively.

### Nicorandil upregulates eNOS expression and downregulates cleaved caspase-3 expression in MCT-injured lungs

**Figure 4:**
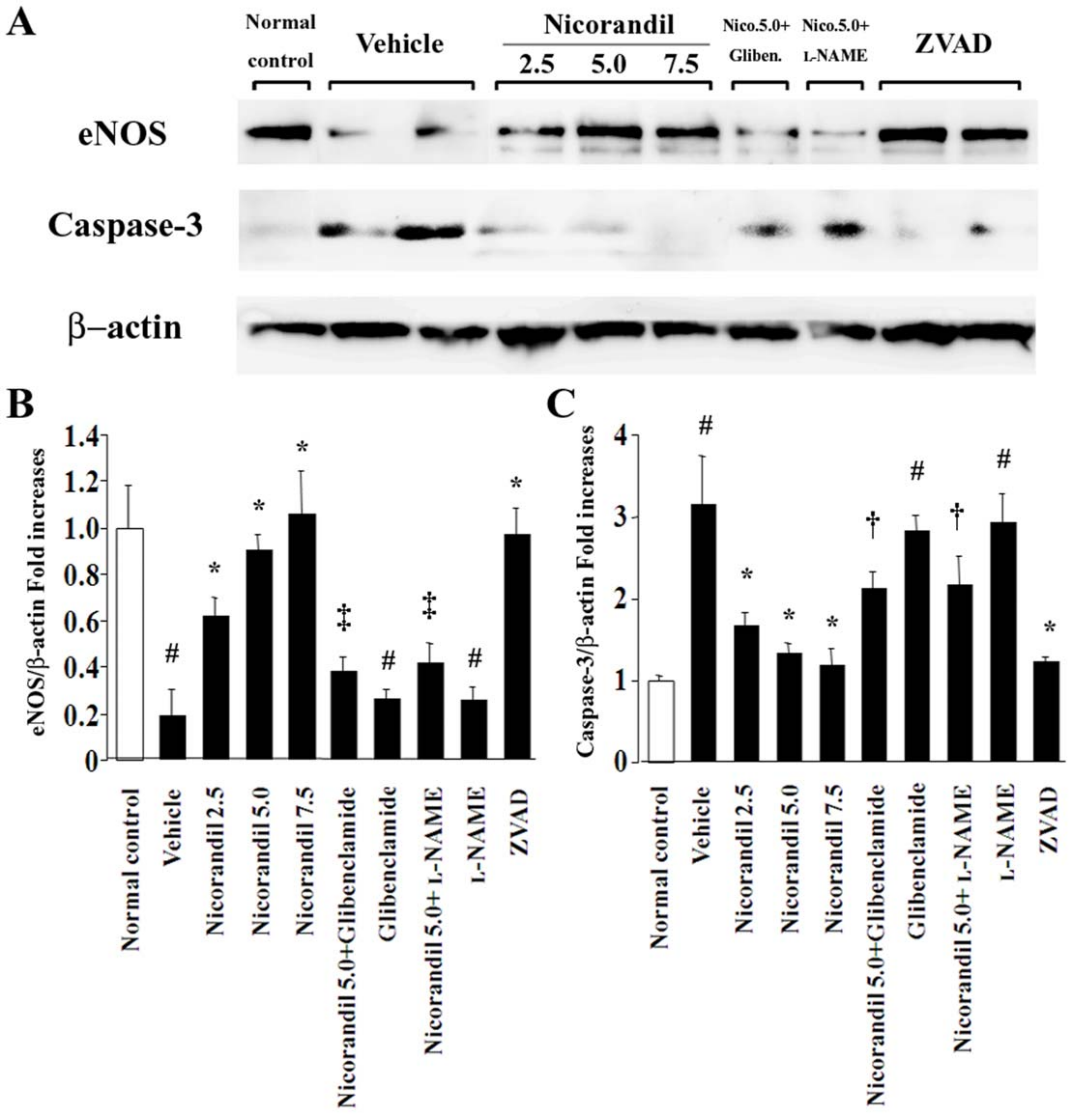
Nicorandil restores the downregulated eNOS expression and attenuates the upregulated caspase-3 expression in MCT-injured lungs. (**A**) The expressions of eNOS and cleaved caspase-3 in lungs were determined by western blotting. MCT significantly downregulated the expression of eNOS and inversely upregulated the expression of cleaved caspase-3 (*see* vehicle). Nicorandil and ZVAD-fmk restored eNOS expression and attenuated caspase-3 expression in MCT-injured lungs, which were blocked by glibenclamide and l-NAME. Bands were quantified using a lumino-analyzer, and the data of eNOS (**B**) and caspase-3 (**C**) expressions were expressed as fold increases normalized to β-actin expression. ^#^ *P*<0.01 vs. normal control; ^*^ *P*<0.05 vs. vehicle; **^†^** *P*<0.05 and **^‡^** *P*<0.01 vs. nicorandil (5.0 mg·kg^−1^·day^−1^).

Western blot analysis revealed that MCT significantly downregulated eNOS expression and inversely upregulated cleaved caspase-3 expression in the lungs (Figure 4A). Notably, nicorandil as well as ZVAD-fmk increased the expression of eNOS and attenuated the expression of cleaved caspase-3 in MCT-injured lungs in a dose-dependent manner, while these effects of nicorandil were also blocked by glibenclamide and l-NAME (Figure 4B and 4C).

### Nicorandil prevents the progression of established PAH

**Figure 5:**
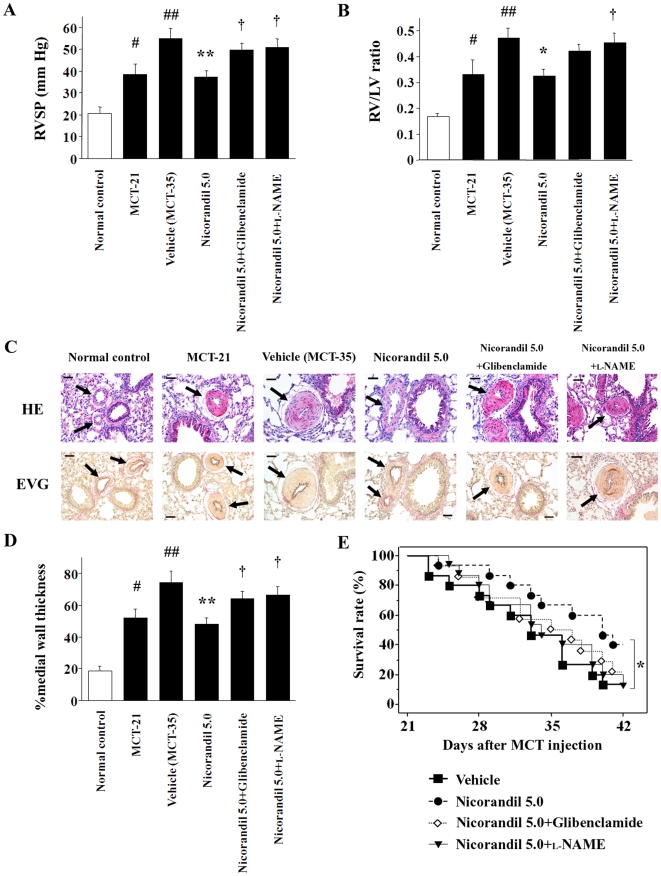
The effects of nicorandil in the reversal protocol. The RVSP (**A**) and the RV/LV ratio (**B**) in the vehicle group increased at 21 days after the MCT injection (MCT-21) as compared to the baseline, and additionally increased in the next 2 weeks (MCT-35). Late treatment with nicorandil on days 21–35 prevented the additional increase in these parameters, while these effects were blocked by glibenclamide and l-NAME. ^#^ *P*<0.05 and ^##^ *P*<0.01 vs. normal control; ^*^ *P*<0.05 and ^**^ *P*<0.01 vs. vehicle (MCT-35); **^†^** *P*<0.05 vs. nicorandil (5.0 mg·kg^−1^·day^−1^). (**C**) The histological findings of the PAs (arrows) in the reversal protocol. Top, HE staining; bottom, EVG staining. Scale bar, 50 µm. (**D**) Late treatment with nicorandil on days 21–35 prevented further increase in the percent medial wall thickness of the PAs. The indications of the symbols are the same as those in panel (A). (**E**) Survival analysis in the reversal protocol. Each group comprised 14–15 rats. ^*^ *P*<0.05 vs. vehicle.

In the reversal protocol, we evaluated whether nicorandil was also effective against established PAH. The RVSP and the RV/LV ratio in the vehicle group increased to 38.7±4.7 mm Hg and 0.33±0.06 at 21 days after the MCT injection (*P*<0.05 vs. the normal control, respectively), and moreover, these parameters increased to 55.0±4.6 mm Hg and 0.47±0.04 at 35 days (*P*<0.01 vs. the normal control, respectively) (Figure 5A and 5B). Late treatment with nicorandil on days 21–35 prevented the additional increases in the RVSP and RV/LV ratio, as the values in the nicorandil-treated group were 37.3±2.9 mm Hg and 0.33±0.03 at day 35 (*P*<0.05 vs. the vehicle, respectively). Histological analysis revealed that nicorandil prevented additional medial wall thickening of the PAs in MCT-injured lungs (Figure 5C and 5D). The survival analysis revealed that nicorandil improved the survival rate in rats with established PAH in the reversal protocol (Figure 5E). The survival rate at 42 days after the MCT injection was 13% in the vehicle group and 40% in the nicorandil-treated group (*P*<0.05). In contrast, these beneficial effects of nicorandil were blocked by glibenclamide and l-NAME, respectively.

### Restoration of eNOS expression by nicorandil in established PAH

**Figure 6:**
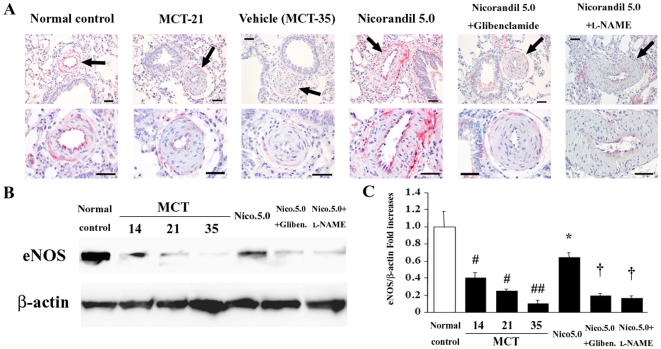
Nicorandil restores eNOS expression in the pulmonary vascular endothelium in established PAH. (**A**) Immunohistochemical staining for eNOS in lung sections in the reversal protocol. Arrows indicate the PAs. Scale bar, 50 µm. (**B**) Detection of eNOS expression in lungs by western blotting in this protocol. MCT downregulated eNOS expression in a time-dependent manner, whereas late treatment with nicorandil resulted in partial restoration of eNOS expression. (**C**) Bands in panel (B) were quantified, and eNOS expression data were expressed as fold increases normalized to β-actin expression. ^#^ *P*<0.01 vs. normal control; ^##^ *P*<0.01 vs. normal control and *P*<0.05 vs. MCT-14; ^*^ *P*<0.01 vs. MCT-35; **^†^** *P*<0.01 vs. nicorandil (5.0 mg·kg^−1^·day^−1^).

In the reversal protocol, MCT downregulated the expression of eNOS in the endothelium of the pulmonary vasculature (Figure 6A) and lung homogenates (Figure 6B and 6C) in a time-dependent manner. Notably, late treatment with nicorandil restored eNOS expression at least partially (Figure 6A–C), while this effect was inhibited by glibenclamide and l-NAME.

### Anti-apoptotic effects of nicorandil on in vitro vascular endothelial cells

**Figure 7:**
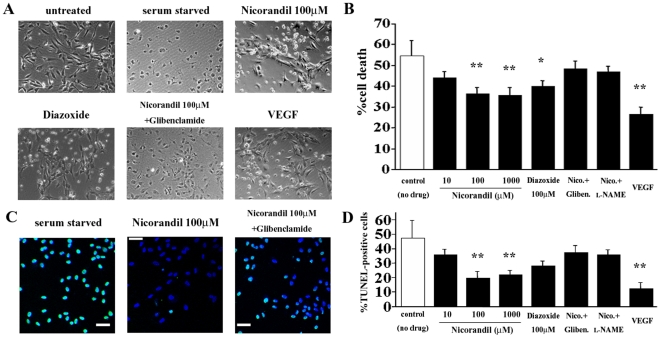
Nicorandil prevents the induction of vascular endothelial cell apoptosis in vitro. (**A**) The HUVECs were incubated in the serum-free medium in the absence or presence of nicorandil (10–1000 µmol/L) for 48 h. The serum-starved HUVECs exhibited apoptotic morphology, which is characterized by cell shrinkage (*see* “serum starved”). (**B**) The viability of the HUVECs was measured with the MTS assay, and the percent cell death was calculated. Stimulation with nicorandil and diazoxide partially restored cell viability in a concentration-dependent manner. VEGF, vascular endothelial growth factor (positive control). ^*^ *P*<0.05 and ^**^ *P*<0.01 vs. control (no drug). (**C**) TUNEL staining revealed that a large number of the serum-starved HUVECs exhibited apoptotic morphology (left), and the apoptotic effects induced by serum starvation were attenuated by nicorandil (middle). TUNEL (green); nuclei (blue). (**D**) The TUNEL-positive nuclei in the serum-starved HUVECs were counted in 10 randomly selected fields and expressed as a percentage of the total number of nuclei. ^**^ *P*<0.01 vs. control (no drug).

Next, we examined the in vitro anti-apoptotic effects of nicorandil for vascular endothelial cells. HUVECs were cultured in serum-free medium and then exhibited apoptotic morphology that was characterized by cell shrinkage (Figure 7A), and there was a decrease in the viability of these cells as determined by the MTS assay (Figure 7B). In addition, there was an increase in the number of serum-starved HUVECs that exhibited apoptotic morphology as identified by TUNEL staining (Figure 7C and 7D). Stimulation with nicorandil and the K_ATP_ channel opener diazoxide partially inhibited the serum starvation-induced endothelial cell apoptosis in a concentration-dependent manner, while these effects of nicorandil were also inhibited by glibenclamide and l-NAME (Figure 7A–D).

### Nicorandil activates the PI3K/Akt and ERK pathways in vascular endothelial cells

**Figure 8:**
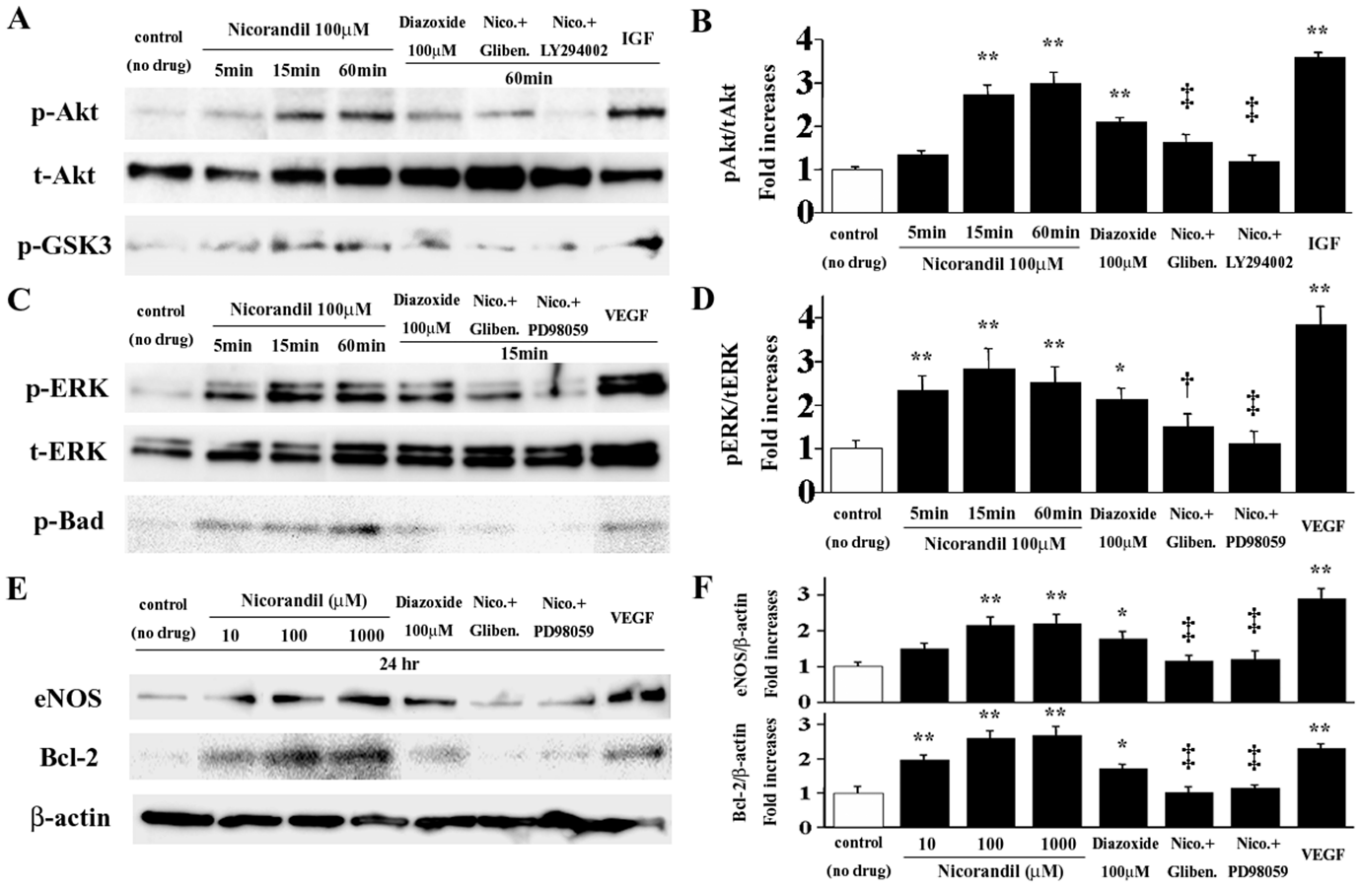
Nicorandil activates the PI3K/Akt and ERK pathways in HUVECs. (**A**) Nicorandil and diazoxide increased Akt serine-473 and GSK-3 phosphorylation in the serum-starved HUVECs, while these effects of nicorandil were blocked by glibenclamide and the PI3K inhibitor LY294002. (**B**) The Akt phosphorylation (p-Akt) data are expressed as fold increases normalized to the total Akt (t-Akt) expression. IGF, insulin-like growth factor (positive control). ^**^ *P*<0.01 vs. control (no drug); **^‡^** *P*<0.01 vs. 60-min nicorandil treatment. (**C**) Nicorandil and diazoxide also increased ERK1/2 threonine-202/204 and Bad serine-112 phosphorylation in the HUVECs, while these effects of nicorandil were blocked by glibenclamide and the MEK inhibitor PD98059. (**D**) The ERK phosphorylation (p-ERK) data are expressed as fold increases normalized to the total ERK (t-ERK) expression. ^*^ *P*<0.05 and ^**^ *P*<0.01 vs. control (no drug); **^†^** *P*<0.05 and **^‡^** *P*<0.01 vs. 15-min nicorandil treatment. (**E**) In serum-starved HUVECs treated with nicorandil for 24 h, the expression of eNOS and Bcl-2 was upregulated in a concentration-dependent manner, while these effects were blocked by glibenclamide, LY294002 (data not shown), and PD98059. (**F**) eNOS and Bcl-2 expression data are expressed as fold increases normalized to the β-actin expression. ^*^ *P*<0.05 and ^**^ *P*<0.01 vs. control (no drug); **^‡^** *P*<0.01 vs. 100-µmol/L nicorandil treatment.

Both the PI3K/Akt and ERK signaling pathways function as cell survival signaling cascades [11], [12]. To investigate the signaling pathways associated with the actions of nicorandil on vascular endothelial cells, we determined the phosphorylation status of Akt and ERK1/2 in the serum-starved HUVECs. Nicorandil (100 µmol/L) induced Akt serine-473 phosphorylation in HUVECs in a time-dependent manner, with a maximum 3.0-fold increase (Figure 8A and 8B). Diazoxide (100 µmol/L) also induced a maximum 2.1-fold increase in Akt phosphorylation. The Akt phosphorylation induced by nicorandil was blocked by glibenclamide (10 µmol/L) and a PI3K inhibitor, LY294002 (10 µmol/L). To determine the Akt kinase activity, we measured the phosphorylation of GSK-3, which is a downstream target of Akt. Nicorandil induced a 2.7-fold increase in GSK-3 phosphorylation (*P*<0.05 vs. baseline), which was blocked by glibenclamide and LY294002 (Figure 8A; bottom). Similarly, nicorandil and diazoxide induced ERK1/2 threonine-202/204 phosphorylation in HUVECs, with a maximum 2.8- and 2.1-fold increase, respectively (Figure 8C and 8D). In addition, serine-112 phosphorylation of Bad, which is a downstream target of ERK1/2, was induced by nicorandil and diazoxide (Figure 8C; bottom), whereas the phosphorylation of ERK1/2 and Bad by nicorandil was blocked by glibenclamide and a MEK inhibitor, PD98059 (10 µmol/L).

Finally, we measured the expressions of eNOS and the apoptosis inhibitor Bcl-2, which is a downstream target of the PI3K/Akt and ERK1/2 pathways, in the serum-starved HUVECs stimulated with nicorandil for 24 h. Notably, nicorandil upregulated the expression of both eNOS and Bcl-2 in a concentration-dependent manner (Figure 8E and 8F), whereas these effects of nicorandil were blocked by glibenclamide, LY294002, and PD98059. Diazoxide also increased the expression of eNOS and Bcl-2, although the degree of the increase induced by diazoxide was lesser than that by nicorandil.

## Discussion

Our experiments revealed that nicorandil attenuated the progression of MCT-induced PAH and improved the survival rate in rats with MCT-induced PAH both in the prevention and reversal protocols. These effects were accompanied by an improvement in the severity of pulmonary vascular remodeling that includes medial wall thickening with the increase of proliferating SMCs, recruitment of macrophages into the perivascular areas, thromboemboli formation in the pulmonary microcirculation, and vascular endothelial cell apoptosis. These effects of nicorandil were closely associated with the enhanced expression of eNOS and anti-apoptotic factors in the vascular endothelium of the lungs, while those were blocked by glibenclamide and l-NAME, suggesting that the beneficial effects of nicorandil on pulmonary vasculature are mediated by the opening of K_ATP_ channels and NOS. In addition, nicorandil induced the activation of the cell survival signaling pathways, PI3K/Akt and ERK1/2, in the vascular endothelial cells, resulting in the production of eNOS and anti-apoptotic factors.

Nicorandil is a nicotinamide ester with 2 vasodilator actions—K_ATP_ channel opening and NO release [8]. The Impact Of Nicorandil in Angina (IONA) study has demonstrated that nicorandil improves the clinical outcome of patients with stable angina [13]. The mechanisms underlying the reduction in major coronary events in the trial are thought to be related to the cardioprotective effects of nicorandil that mimic ischemic preconditioning through K_ATP_ channels. Although the effects of nicorandil in PAH have remained undetermined, a few reports based on animal studies [14] and clinical experience [15] have suggested its potential efficacy for PAH treatment. In contrast to our results, Hongo et al. [14] reported that late treatment with nicorandil could not prevent further development of PAH nor prolong survival in rats with established PAH. The reason for this discrepancy remains unclear, but it may be attributable to the difference in the injection method of nicorandil (drinking water or subcutaneous osmotic pumps).

MCT, a pyrrolizidine alkaloid toxin, induces selective pulmonary endothelial injury and apoptosis, followed by severe inflammatory responses and medial hypertrophy [6], [16]. In our study, nicorandil protected the endothelium of the pulmonary vasculature from MCT injury by both restoring eNOS expression and inhibiting the induction of endothelial cell apoptosis in vivo and in vitro. Cumulative evidence suggests that endothelial cell apoptosis in pulmonary vasculature might trigger pathological vascular remodeling, leading to the progression of PAH [4]–[7], [10]. Consistent with the findings of a previous report [5], the broad caspase inhibitor ZVAD-fmk also attenuated the development of MCT-induced PAH in the present study, accompanied with a decrease in endothelial cell apoptosis in the pulmonary microvasculature. It is plausible that the anti-apoptotic effects of nicorandil and ZVAD-fmk on the pulmonary vascular endothelium might contribute to the blocking of development of pathological pulmonary vascular remodeling induced by MCT and therefore lead to the improvements on pulmonary hemodynamics and survival in rats with MCT-induced PAH. On the other hand, medial hypertrophy itself by proliferation of vascular SMCs is also a major histopathological finding in pulmonary vasculature under PAH [1]–[3], and nicorandil seems to have the direct and/or indirect effects on SMCs in the media of PAs as nicorandil markedly reduced the number of proliferating SMCs (Figure 3). Our results are consistent with the report demonstrating that nicorandil has anti-proliferation effects on rat aortic SMCs [17]. Intriguingly, a specific anti-apoptotic reagent ZVAD-fmk also attenuated proliferation of medial SMCs in the remodeled PAs in the similar manner as nicorandil. Since we found no TUNEL-positive apoptotic cells among proliferating medial SMCs in PAs of the MCT-injured lungs (Figure 2D–E), it would appear that ZVAD-fmk exerted anti-apoptotic effects on pulmonary vascular endothelial cells, not on medial SMCs, in this setting, resulting in the indirect anti-proliferation effects on medial SMCs. Taken together, nicorandil might attenuate medial hypertrophy of PAs in this model by not only the direct effect on PA-SMCs but also the indirect effect through blocking the induction of apoptosis on the pulmonary vascular endothelium. This notion is supported by the results of the recent report that has revealed that the absence of normal immune regulation results in an inappropriately exuberant inflammatory response and accelerated endothelial cell apoptosis, leading to smooth muscle hypertrophy and increased pulmonary vascular resistance [18].

Although the anti-apoptotic effects of nicorandil on vascular endothelial cells have not been reported so far, nicorandil is known to exert anti-apoptotic effects on cardiomyocytes via the activation of K_ATP_ channels [19]. The protective effects of nicorandil on the pulmonary endothelium in our study were involved in the eNOS production, and this finding is consistent with a previous study [20] that revealed that nicorandil enhanced eNOS expression in the myocardium via the opening of K_ATP_ channels. In our study, the K_ATP_ channel closer glibenclamide inhibited the nicorandil's protective effects, including the enhanced expression of eNOS on the pulmonary endothelium, suggesting that eNOS production induced by nicorandil appeared to be a downstream event after the opening of K_ATP_ channels. The notion is supported by the findings of a previous report [21] that showed that a K_ATP_ channel opener protected the myocardium against lethal ischemia via NOS production.

The production of anti-apoptotic factors and eNOS by nicorandil was associated with the activation of both the PI3K/Akt and ERK1/2 signaling pathways. Indeed, the PI3K/Akt and ERK1/2 signaling pathways play a crucial role in cell survival and regulation of apoptosis [11], [12]. Many downstream effectors in the PI3K/Akt and ERK1/2 signaling pathways, including Bcl-2, Bad, and the caspase family members, function as inhibitors of apoptosis. Intriguingly, the ERK1/2 pathway has been reported to undergo activation in response to reactive oxygen species (ROS) [22], and the opening of mitochondrial K_ATP_ channels has been shown to generate mitochondria-derived ROS in cardiomyocytes [23]. Thus, owing to its K_ATP_ channel-opening property, nicorandil may activate the ERK1/2 pathway via the generation of ROS in the mitochondria in vascular endothelial cells, although not addressed in this study; the notion is supported by evidence from the literature that has shown that another K_ATP_ channel opener diazoxide also triggers ERK activation through mitochondria-derived ROS in cardiomyocytes [24]. On the contrary, the opening of mitochondrial K_ATP_ channels has been reported to activate the PI3K/Akt pathway in the cardiomyocytes of rodents as well [21], [25]. In addition, NO has been shown to induce the activation of both the PI3K/Akt and ERK1/2 signaling pathways through a cyclic guanosine monophosphate-dependent pathway [24], [26]. Taking into consideration that the degree of the in vitro effects induced by diazoxide in our study was lesser than that induced by nicorandil, not only the K_ATP_ channel-opening effect but also the NO-releasing property itself of nicorandil may contribute to the activation of the PI3K/Akt and ERK1/2 pathways, resulting in the beneficial protective effects against serum starvation in vitro and MCT injury in vivo.

NO is known to dilate the pulmonary vessels and used in the short-term treatment of patients with severe PAH derived from a variety of origins, although there is limited experience with the long-term use of inhaled NO as a treatment of PAH [1], [2], [27]. In the present study, a slow NO-releasing drug, sr-ISDN, also attenuated MCT-induced PAH in rats to some degree; however, the effects without changing systemic blood pressure and heart rate were modest compared with nicorandil. Given that a K_ATP_ channel closer glibenclamide inhibited approximately 60–70% of the nicorandil's protective effects for deteriorated pulmonary vasculature and hemodynamics in rats with MCT-induced PAH, it seems that the major beneficial effects of nicorandil are attributed to its K_ATP_ channel opening property, while its NO donor property functions adjunctively in this setting. Alternatively, the feature as being a unique hybrid drug with the 2 vasodilator actions may be a crucial advantage of nicorandil, at least in the context of treatment of PAH.

In conclusion, the present study has revealed that nicorandil attenuates MCT-induced vascular endothelial damage and apoptosis and PAH through the production of eNOS and anti-apoptotic factors, which is mediated by the PI3K/Akt and ERK1/2 signaling pathways. These results strongly supports the notion that nicorandil has a promising therapeutic potential for PAH.
